# Managing Stress and Somatization Symptoms Among Students in Demanding Academic Healthcare Environments

**DOI:** 10.3390/healthcare12242522

**Published:** 2024-12-13

**Authors:** Maria Antoniadou, Georgia Manta, Antonia Kanellopoulou, Theodora Kalogerakou, Alessandra Satta, Polyxeni Mangoulia

**Affiliations:** 1 Department of Dentistry, School of Health Sciences, National and Kapodistrian University of Athens, 11527 Athens, Greece; akanel@dent.uoa.gr (A.K.); tkalogerakou@hotmail.com (T.K.); alessandra.satta.be@gmail.com (A.S.); 2 Certified Systemic Analyst Program (CSAP), Systemic Management, University of Piraeus, 18435 Piraeus, Greece; 3 Faculty of Nursing, School of Health Sciences, National and Kapodistrian University of Athens, 11527 Athens, Greece; gmanta@nurs.uoa.gr; 4 Instituut voor Kern-en Stralingsfysica, Department of Physics, KU Leuven, 3001 Leuven, Belgium

**Keywords:** stress somatization, nursing students, dentistry students, somatization symptoms, coping strategies, academic stress, stress management, depression, psychological support, mentoring, coach

## Abstract

Introduction: Stress is a common concern among healthcare students, due to the demands of their coursework and the elevated expectations they face. Especially among dentistry and nursing students, the phenomenon, although well-documented, covers psychosocial and physiological dimensions, with somatization symptoms being less explored. These manifestations are crucial to identify discipline-specific stressors and health impacts that can lead to targeted interventions for both disciplines. Aim: This study investigates stress perceptions, somatization, and coping strategies among 271 nursing and dentistry students at the National and Kapodistrian University of Athens. Methodology: An e-questionnaire was open for submissions during February and March 2024. Results: Females reported higher stress somatization (M = 10.22, SD = 5.23) than males (M = 7.94, SD = 6.14; Cohen’s d = 0.412, *p* < 0.05). The interpretation of stress as “restlessness and psychological pressure” was more prevalent in dentistry students compared to nursing students. Moreover, nursing students who perceived stress as the “inability to manage unexpected or difficult situations, insecurity, panic” were more likely to experience stress somatization symptoms, while for dentistry students, stress somatization was related to “pressure to meet daily obligations/long-term goals”. Physical symptoms for all students included chest discomfort, digestive issues, and headaches/nausea. Also, dentistry students reported more teeth clenching or grinding than nursing students. Short-term coping strategies included emotional balance, managing stressors, situation analysis, and breathing techniques. Long-term strategies involved distraction and entertainment, physical exercise, and patience. A higher willingness to seek coaching support correlated with higher stress somatization among dental students. Nursing students favored psychological support, while dentistry students suggested curriculum revision and improved infrastructure. Discussion/Conclusions: Females exhibited higher stress somatization levels, with themes of insecurity and physical symptoms. Nursing students reported higher somatization linked to insecurity, while dental students associated stress with daily obligations and goals. The study highlights the need for improved support systems, flexible academic procedures, and better communication to address stress in healthcare academia.

## 1. Introduction

Stress is a prevalent issue among healthcare students, arising from the demanding nature of their studies and the high expectations placed upon them [1]. The phenomenon of stress has been studied within each healthcare discipline, addressing various psycho-social and physiological dimensions [2,3]. However, there is a notable lack of comparative studies [3], particularly between dentistry and nursing students, to allow for a detailed exploration of the unique stressors specific to these two disciplines, which can differ significantly from those encountered in general medical training [4,5].

From the relevant literature, we know that the educational demands of both dentistry and nursing disciplines that contribute the most to student stress and require special attention include clinical performance pressure, extensive practical experience, high patient interaction, rigorous coursework, shift work, and rotations, and high expectations for professional competence [5]. Dentistry students, in particular, face immense pressure to develop precise manual skills and perform clinical procedures accurately under time constraints [6]. The high expectations placed on them by their academic institutions and future professional roles add to their stress as they work to meet these standards during their studies [7]. Further, nursing students often experience stress due to irregular hours and shift work during clinical rotations, which disrupts their work-life balance and adds to their anxiety [8]. Obviously, in both disciplines, students must complete numerous clinical hours, often balancing this with heavy coursework [8]. Constant patient care responsibilities add to the stress as they must manage patient therapies and well-being with limited experience [1,2,3,7,8,9]. It is finally reported that both disciplines demand mastery of a vast amount of complex information, which can overwhelm students and increase their stress levels [10].

While psychological aspects of stress have been extensively studied [11,12], the physical manifestations—such as unexplained physical symptoms arising from psychological distress—have received comparatively less focus [13,14]. This study aims to explore the phenomenon of stress somatization in these student groups to show how psychological distress can manifest as physical symptoms without any identifiable organic cause [15]. Understanding these somatic expressions of stress is crucial, as they may affect students’ health and their capacity to perform in high-stakes clinical environments [16]. In this study, we aim to contribute to a more comprehensive understanding of stress somatization in healthcare education.

Among dentistry students, somatization symptoms such as tension-type headaches with a positive association with painful temporomandibular disorders (TMD) or bruxism are prevalent, linking these symptoms to stress and psychological functioning [17]. Research highlights the association between emotional stress and awake bruxism, with sleep disturbances also playing a role [18]. Awake bruxism, characterized by clenching and grinding teeth during waking hours, is linked to psychological factors and perceived stress levels [17,19,20]. On the other hand, sleep bruxism has been correlated with stress and depression [21,22,23,24,25]. Also, demographic factors such as gender and age may additionally influence the experience of stress and somatization symptoms among dentistry students [26,27]. More specifically, gender differences in stress response are evident, with females often reporting higher levels of stress-related symptoms compared to males, suggesting gender socialization plays a role in stress responses [28]. Age-related changes in proprioceptive function and fine motor skills contribute to the variation in how stress manifests somatically among dentistry students [29]. Typically, first-year dentistry students experience higher levels of stress due to the transition from theoretical learning to practical applications, which includes the development of precise manual skills and patient care responsibilities [24]. Additionally, the increased academic and clinical demands during the later years, particularly in the fourth and fifth clinical years, also contribute to heightened stress levels as students prepare for professional practice and face more complex clinical cases [30]. This stress can lead dentistry students to somatization in different parts of the body, where psychological distress is expressed through various physical symptoms [31].

Similarly, nursing students are not immune to the somatic effects of stress, with studies highlighting associations between stress and physical health outcomes [32]. Nursing students also encounter substantial stressors throughout their education, which can lead to somatization of stress and various physical health issues [33,34]. Recent studies discuss the prevalence and risk factors associated with stress among nursing students, highlighting the need for targeted interventions to support their well-being [35,36,37]. Factors such as heavy workloads, academic pressure, and exposure to emotionally challenging situations during clinical placements contribute to the psychological distress experienced by nursing students [38]. This distress is expressed through physical health problems, too [39]. Additionally, the demanding nature of nursing education and clinical practice can negatively affect physical health conditions, further highlighting the importance of holistic support for them [40]. It is reported that sleep disturbances, musculoskeletal problems, and gastrointestinal issues are among the common somatic manifestations of stress reported by nursing students [41]. Moreover, the experience of stress-induced somatic symptoms may vary based on demographic factors such as gender, age, and academic year [26,27,42]. For example, female nursing students tend to report higher levels of stress-related somatic symptoms compared to males, potentially influenced by gender-specific stressors and coping mechanisms [43,44,45]. Additionally, younger students and those in earlier academic years may be more susceptible to stress-related somatic symptoms due to academic adjustments and their first approach to clinical practice demands [44,45,46,47,48].

Furthermore, coping strategies are pivotal for managing stress among dentistry and nursing students, given the demanding educational environments they face. Common coping strategies employed by students include problem-focused approaches, such as time management and seeking academic support, aimed at directly addressing stressors [49,50]. Emotion-focused coping strategies, such as mindfulness and seeking social support, also help manage the emotional responses to stress [51]. Conversely, it is reported that avoidance coping strategies, like procrastination or denial, may provide temporary relief but can exacerbate stress over time [52]. It seems, though, that effective coping strategies are crucial for enhancing resilience, improving academic performance, and reducing negative outcomes such as anxiety and depression among students [53,54]. So, the unique stressors in dentistry and nursing education support the necessity of incorporating adaptive coping mechanisms to enhance students’ well-being and professional development [55].

While the phenomenon of stress among healthcare students is well-documented, the idea that stress experiences and somatic manifestations are largely similar across disciplines is not fully supported by the evidence. Second, while general studies on healthcare students provide a foundational understanding of stress, they do not adequately account for the distinct academic, clinical, and emotional stressors faced by students in specific programs. So, the lack of comparative studies is limiting our understanding of how these stressors and their somatic effects differ between healthcare disciplines such as dentistry and nursing. These discipline-specific stress responses, coupled with demographic influences such as gender and age, prove the inadequacy of a one-size-fits-all approach to managing stress among healthcare students. Moreover, we are now living in a VUCA (Volatile, Uncertain, Complex, and Ambiguous) world [56], especially influenced by the recent pandemic [57], which has fundamentally altered the educational and professional landscape. The unprecedented challenges faced in this new reality show us the need to study various healthcare disciplines in isolation and conjunction to better understand their unique stressors and coping mechanisms [46]. This understanding is vital to developing educational activities, curriculum revisions, and innovative approaches that are responsive to these evolving demands [54]. Thus, more focused research is needed to provide tailored interventions that promote resilience, reduce stress-induced symptoms, and enhance both the academic performance and professional development of healthcare students. Therefore, the current study focuses on a pilot stage, to explore these discipline-specific stressors and discuss the theme of somatization of these phenomena to assess students’ needs for “VUCA”-derived tailored interventions in the fields of academic healthcare. Ultimately, this study aims to provide valuable insights that can lead to targeted interventions and support initiatives planning to enhance the well-being of dentistry and nursing students.

The null hypothesis for the study is that there is no significant difference in the prevalence of somatization symptoms, perceived stress levels, coping strategies, resilience factors, academic performance, and overall well-being between dentistry and nursing students.

## 2. Materials and Methods

### 2.1. Design of the Study

This study is part of a larger project that included a systematic review, a questionnaire-based study using multiple tools to estimate stress prevalence, and structured interviews. In this study, we focus on the data collection related to stress somatization. The present concurrent mixed-methods study based on a questionnaire formation and use integrates both quantitative and qualitative approaches to comprehensively explore stress perceptions, coping strategies, and resilience among dentistry and nursing students [58,59]. The study was conducted in three phases.

We first employed an extensive literature review focused on stress, somatization, and coping strategies among healthcare students. This review allowed us to gather relevant information from various studies and to identify key concepts and recurring themes in the existing literature. While this review was comprehensive, it was not conducted as a formal systematic review. Instead, the primary purpose of this process was to inform the development of a relevant and targeted questionnaire, which aimed to explore stress somatization specifically among dentistry and nursing students. So, by doing this, we formed the primary questionnaire for our study. To ensure its content validity, a panel of experts in psychology, nursing, and dentistry from the two departments reviewed the items for relevance and clarity, refining them as needed [60]. A pilot study was then conducted with 10 students from each department (10 undergraduate and 10 postgraduate) who voluntarily agreed to participate to assess comprehensibility and identify ambiguities [61]. This feedback led to further refinement, ensuring the questionnaire’s applicability. The final questionnaire employed underwent a thorough validation process to ensure its reliability and validity [62,63]. Construct validity was confirmed through exploratory factor analysis (EFA), verifying that the questionnaire effectively measures the intended constructs [64]. Reliability was also tested, with Cronbach’s alpha coefficients showing high internal consistency (at 0.750), well above the recommended threshold of 0.70 [65].

### 2.2. Ethical Approval and Confidentiality of Data

Ethical approval was obtained from the respective ethics committees to ensure uniform participation. More specifically, the study was conducted following the Declaration of Helsinki and approved by the Institutional Review Board (or Ethics Committee) of the Department of Dentistry (609/3 October 2023) and Nursing (467/26 October 2023) of the School of Health Sciences of the National and Kapodistrian University of Athens, Greece.

In the main study phase, all participants were provided with detailed information about the study’s purpose, procedures, potential risks such as emotional discomfort, and benefits. Informed consent was obtained from each participant, ensuring their voluntary participation.

Confidentiality was maintained through the anonymization of data, safeguarding participants’ privacy and minimizing the risk of stigma associated with sensitive health information. Data security protocols were strictly followed to prevent unauthorized access. Respect for autonomy was upheld by allowing participants the right to withdraw from the study at any time without repercussions. Finally, findings have been disseminated accurately and transparently to contribute to knowledge in the field while respecting the participants’ contributions and privacy.

### 2.3. Population, Sample, Sample Size and Sampling Technique

The study recruited a sample of undergraduate (N1A = 780) and postgraduate students from the Department of Dentistry (N1A = 85) and Nursing (N1B = 808, N2B = 330) at the School of Health Sciences, National and Kapodistrian University of Athens, Greece. The sampling procedure for this study was designed to ensure comprehensive participation and representation of both undergraduate and postgraduate students from the Departments of Dentistry and Nursing at the School of Health Sciences, National and Kapodistrian University of Athens. Lists of eligible students were obtained through the secretariats of the respective departments to create an inclusive and systematic distribution strategy. The questionnaire link was disseminated electronically via institutional email addresses, ensuring accessibility and minimizing the potential for excluding participants. Electronic distribution was chosen due to its broad reach, convenience, and ability to accommodate diverse student schedules. Additionally, to optimize participation, the survey link was made available for two months between February and March 2024, with reminders sent every 15 days. These reminders, sent to all eligible students, emphasized the voluntary and anonymous nature of the study, the study’s objectives, and the submission deadline to encourage participation while maintaining a non-coercive approach.

The questionnaire was designed specifically for this study and was uploaded to Google Forms, which generated a QR code for added accessibility. To accommodate participants who preferred offline sharing, the QR code was distributed through lecture announcements and posted in common areas such as departmental notice boards. To enhance usability and inclusivity, the survey link and QR code were tested on multiple devices and platforms to ensure compatibility. The design of the questionnaire underwent a strict development process, drawing upon existing validated tools and adapting them to the study’s specific objectives. Although the study did not conduct a formal pretest, internal reviews were performed to identify and address ambiguities, ensuring clarity and relevance of the questions.

Ethical considerations were prioritized throughout the study. Approval was obtained from the institutional ethics board, and the research adhered to the principles outlined in the Declaration of Helsinki. Anonymity was strictly maintained by disabling the collection of IP addresses and securely storing responses on password-protected systems accessible only to the research team. Participants were assured of confidentiality, and no identifiable personal data were collected, as stated explicitly in the study instructions. The survey system was configured to allow only one submission per participant, minimizing duplicate responses and preserving the integrity of the data.

Inclusion criteria for the study required active enrollment in undergraduate or postgraduate programs within the departments of dentistry or nursing. Students on academic leave or those outside these specific programs were excluded from participation. The study aimed to capture a representative sample of the student population by accounting for varying academic schedules and ensuring participation was feasible during the study period, which was carefully selected to avoid major examination periods or academic holidays.

To monitor the quality and progress of data collection, submission rates were tracked regularly. Any technical issues reported by participants were promptly addressed to prevent disruptions in response collection. At the end of the survey period, response rates were calculated separately for each department and program (undergraduate and postgraduate) to assess representativeness. While no formal compensation was provided, participants were regularly reminded of the significance of their contributions to the study’s objectives to enhance engagement and commitment to completing the questionnaire.

### 2.4. Study Instrument

The final study e-questionnaire consisted of three distinct sections: (1) Part One (Q1–Q7: questions related to demographic characteristics), (2) Part Two (Q8–Q14: questions related to symptoms concerning the stomatognathic system due to stress), and (3) Part Three (Q15–Q22: includes open-ended questions aimed at gathering desired actions and initiatives intended for the management and handling of stress, as well as the improvement of resilience) [66,67,68]. The questionnaire was carefully designed to link the somatic symptoms reported by participants to their experiences of stress. We addressed this issue by the following facts: (1) the questionnaire items on somatization were developed based on an extensive review of existing literature, which highlights the well-documented connection between stress and somatic symptoms in healthcare students. We then selected items that are commonly associated with stress-induced physical symptoms. (2) Participants were asked to reflect specifically on stress-related physical symptoms. The questionnaire prompted them to consider somatization concerning their experiences of stress, ensuring that the symptoms they reported were understood as being stress-related rather than due to other causes. (3) The open-ended questions allowed participants to describe their stress experiences in detail, which helped us validate the connection between stress and the somatization symptoms they reported. Thematic analysis of these responses further supported the link between stress and somatization. The questionnaire of the study and guidelines for fulfillment are presented in Appendix A and Appendix B.

### 2.5. Data Collection

The quantitative component of the study involved the use of a structured questionnaire designed to gather demographic data and measure stress somatization. The quantitative data were analyzed using descriptive statistics, *t*-tests, and Spearman correlation coefficients to explore relationships and differences in stress somatization between genders and departments. In parallel, the qualitative component consisted of open-ended questions in the same questionnaire, analyzed using thematic analysis to identify recurring themes and patterns in participants’ responses regarding stress manifestations, coping strategies, and suggestions for improvement [69].

In the first and second phases of the study, both types of data were collected simultaneously, allowing for a complementary approach. The quantitative data provided broad, measurable trends, while the qualitative responses offered deeper context and personal experiences, enriching the initial understanding [9,21,33,38]. The integration of both data types in the third study phase allowed for a richer and deeper interpretation of our results, offering a more holistic understanding of stress experiences and coping mechanisms in healthcare students, balancing both numerical data and personal narratives. Responses were coded independently by two researchers to ensure reliability, and discrepancies were resolved through discussion to achieve consensus.

### 2.6. Data Analysis

Data analysis was then performed with the statistical package IBM SPSS v. 28. Data for demographics and the stress somatization scale were analyzed by producing descriptive statistics. Stress somatization distribution was examined in terms of normality via skewness and kurtosis and was considered normally distributed [70]. Therefore, independent samples t-tests were used to assess differences in stress somatization between genders and departments. Qualitative data collected from open-ended questions were analyzed thematically, and absolute and relative frequencies (n, %) were reported per theme/category and question. Potential differences in the theme/categories extracted between students’ genders and the department of study were assessed with chi-square tests of independence performed with Fisher exact test correction when needed [71]. Moreover, correlations of theme/category prevalence and stress somatization levels were examined with the non-parametric Spearman correlation coefficient.

Once both data types were analyzed, the results were combined to form a comprehensive understanding of the student’s experiences with stress and coping mechanisms. Quantitative data provided the “what”, showing which groups were more affected by stress, while qualitative data explained the “why” and “how” by shedding light on the personal experiences and contextual factors that influenced those results. This integrated approach allowed the study to balance objective data with the richness of personal experiences, providing a fuller picture of stress somatization and coping strategies among dentistry and nursing students. For instance, if certain groups showed higher stress levels quantitatively, the qualitative data might reveal the underlying reasons or coping mechanisms used by these students. So, we had a richer, multi-layered interpretation of our findings.

Furthermore, in our study, the qualitative data collected from the open-ended responses were processed systematically through the following steps: (1) All open-ended responses were transcribed verbatim to ensure that the participants’ exact words and expressions were captured; (2) Two researchers independently reviewed the transcriptions and applied open coding to the data. During this stage, keywords, phrases, or ideas related to stress manifestations and coping strategies were identified. These codes represented initial concepts without any predefined categories, allowing the data to speak for itself; (3) After initial coding, the researchers compared their codes and discussed any discrepancies to ensure consistency. Related codes were then grouped into broader categories. For example, codes related to physical symptoms, emotional responses, or specific coping mechanisms were grouped into distinct categories. This step helped organize the data into manageable clusters, and (4) Once the categories were established, the researchers identified overarching themes that emerged from the data. These themes represented higher-level concepts that captured the essence of the participants’ experiences.

More specifically, during the qualitative analysis, the first step was to transcribe the interviews verbatim. Each transcript was reviewed multiple times by the research team to ensure familiarity with the content. Initial codes were developed based on recurring phrases or concepts that were relevant to stress somatization, coping strategies, and resilience. For instance, a common code identified in the interviews was “emotional exhaustion”, which reflected students’ descriptions of being mentally drained by their academic workloads. These initial codes were grouped into broader themes. For example, codes related to physical symptoms like “neck pain”, “headaches”, and “fatigue” were grouped under the theme “Somatization of Stress”, while references to emotional support, advice from friends, and coping mechanisms through relaxation were categorized under the theme “Coping Strategies and Social Support”. Also, themes such as “physical manifestations of stress”, “emotional coping mechanisms”, and “institutional support for stress management” were derived accordingly from this categorization process. Throughout this process, the researchers engaged in regular discussions to refine the themes and ensure that those accurately reflected the data. Overall, the development of these themes was a collaborative process. After initial coding, the team met regularly to review the codes and refine the themes. Discrepancies in coding were resolved through discussion, and final themes were defined and validated by revisiting the raw data. The final themes were supported by verbatim quotes from the participants, which helped illustrate how the themes emerged directly from the data.

Finally, we should mention that the quantitative data (e.g., stress somatization scores) were analyzed using descriptive statistics and Spearman correlations to assess relationships and differences between variables such as gender and department. The qualitative data collected through open-ended responses were analyzed separately using thematic analysis to identify recurring themes and patterns in students’ experiences with stress and coping strategies. So, while the quantitative and qualitative findings were integrated to provide a comprehensive understanding of stress and somatization, the qualitative insights enriched the interpretation of the quantitative results but were not subjected to statistical testing.

## 3. Results

### 3.1. Demographic Profile

Analysis revealed that for stress somatization, Cronbach’s alpha is 0.750. The sample consisted of 271 students pursuing their undergraduate or postgraduate degrees in the nursing department (n = 126, 46.5%) or the dentistry department (n = 145, 53.5%). The female gender was more prevalent (68.60% female students), while the majority, 80.10%, were undergraduate students, with 52.20% reporting a family income below EUR 25,000 (Table 1).

### 3.2. Analysis of Data per Gender and Department

As presented in Table 2, dentistry students had higher rates (41%) compared to nursing students (28.20%) in terms of somatization symptoms. Also, there was a statistically significant, moderate difference between male and female participants in stress somatization (*p* < 0.05, Cohen’s d = 0.412), with female students presenting increased levels of stress somatization (M = 10.22, SD = 5.23) compared to male students (M = 7.94, SD = 6.14) with dentistry students reporting more often on the subject than nursing students but at a non-statistically significant level. There were no statistically significant effects of educational level, year of studies, or family income on stress somatization.

**Figure 1:**
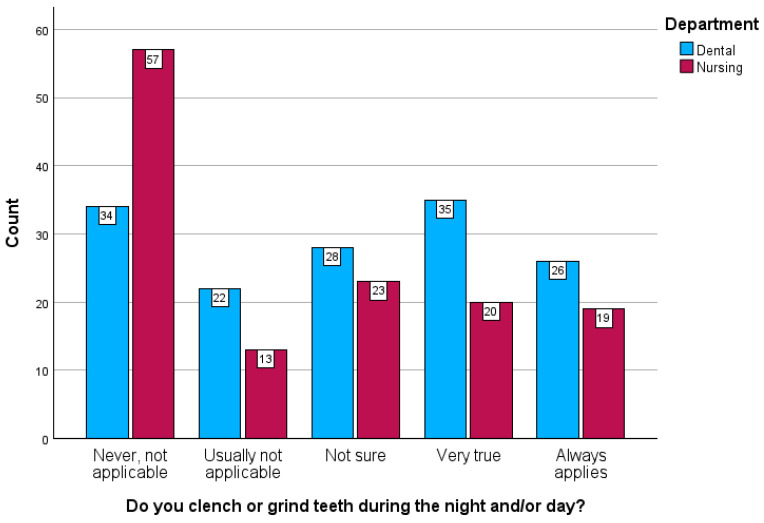
Clustered bar chart of clenching or grinding teeth during the day or night by the department.

Breaking down the stress somatization to its seven items (symptoms) (Figure 1), female participants reported hearing a sound from the temporomandibular joint during movements of the mandible (very true or always applicable) at a higher rate of 24.8% in contrast to 15.1% of male participants (*p* = 0.039). Also, female participants reported a percentage of 43.1% that they experienced headaches, compared to a smaller ratio of 18.6% for male participants (*p* < 0.001). Finally, female participants reported 35.8% that they feel nervous or annoyed at their relationships in the student/work environment, compared to a smaller ratio of 26.7% for male participants (*p* < 0.001).

Moreover, dentistry students reported that they experienced clenching or grinding teeth during the day or night (42.7%), compared to a smaller ratio of 29.5% for nursing students (*p* = 0.010). There were no statistically significant effects of educational level, year of studies, or family income, neither on the composite stress somatization scale nor its respective items (symptoms) (Table 3).

Students’ responses to the open-ended questions were categorized based on their content relative to perceptions of stress, stress management strategies, and requirements. Table 4 presents the categories/themes extracted from the qualitative analysis by students’ department (nursing/dentistry) and gender (male/female).

In summary, the combination of quantitative and qualitative findings in our study reveals that stress somatization is prevalent among healthcare students, with notable gender differences. Female students reported higher rates of headaches, nervousness, and somatic symptoms, which qualitative data suggest may be related to emotional stressors such as relationship difficulties and academic pressures. Conversely, dentistry students reported higher instances of teeth clenching, which qualitative interviews linked to the high demands of their clinical training. Together, these findings show us the importance of addressing both the physical and emotional components of stress through targeted support interventions tailored to the unique needs of different student groups.

### 3.3. Understanding the Meaning of Stress by Students (What Does “Stress” Mean to You?)

In the question about their interpretation of stress, the category “*Inability to manage unexpected or difficult situations, Insecurity, Panic*” gathered the largest percentage of responses (38.38%), which includes the insecurity, fear, or panic caused by unexpected or difficult situations that are either happening or may happen in the future. Following, the second most frequent category was the one of “*Restlessness and psychological pressure*” (34.32%) and the category of “*Physical Symptoms*” manifested due to anxiety (19.93%) such as tachycardia, head and chest pains, stomach disorders, fatigue, etc.

A significant number of students explained stress as the difficulty in concentration and the disorganization of the person’s thoughts and feelings (17.71%) which indicates the person’s sudden reduced ability to concentrate, think productively, and express their feelings. The remaining manifestations of anxiety include obsessive thoughts and overanalyzing (9.59%), the daily pressure of the individual to meet his obligations combined with the pressure of long-term goals (9.59%), and finally the fear of failure (6.64%).

The interpretation of stress as “*Restlessness and psychological pressure*” was more prevalent in dentistry students compared to nursing students (*p* < 0.05, 41.0% vs. 28.2%, respectively, see Table 4). Moreover, nursing students who perceived stress as the “*Inability to manage unexpected or difficult situations, Insecurity, Panic*” were more likely to experience stress somatization symptoms (ρ = 0.276, *p* < 0.05), while for dentistry students, stress somatization was related to “*Pressure to meet daily obligations/long-term goals*” (ρ = 0.186, *p* < 0.05).

### 3.4. Stress Somatization Symptoms (How and Where in the Body Do You Experience Stress?)

Regarding the physical symptoms with which anxiety manifests itself in students, the most common of them are heart and chest discomfort (41.8%), such as tachycardia and a feeling of heaviness in the chest, as well as digestive/nutritional disorders (41.41%) such as stomach pains, ulcers, constipation, anorexia, etc. Followed by headaches (36.33%), breathing difficulties (11.72%), general muscle tension (10.94%), such as tremors in the hands and the legs and sweating, or various skin diseases (10.55%). There were no significant differences between male and female participants or between dentistry and nursing departments in the categories extracted for body manifestation of stress (see Table 3 and Table 5).

### 3.5. Attitude Towards Resilience Building by Students (How Do You Feel You Can Improve Your Resilience?)

In terms of the ways that students believe can help them improve their resilience to stress, the highest percentage of common acceptance is gathered by “*Emotional balance and Self-awareness*” (34.44%), a category that generally includes any effort to develop and improve one’s identity. This is followed by “*Managing Stressors*” with an acceptance rate of 25.93%, which mainly includes voluntary exposure to uncertainty to gain experience in dealing with problems as well as other stress reduction strategies. Lower down, we see the category of “*Psychotherapy and Psychological support*” (17.41%) and immediately after the category of “*Improving Physical Condition, Nutrition and Sleep*” (10.74%). In addition, other methods of reducing stress include “*better time management*” (9.26%), “*seeking social support*” (7.41%), and “*devoting oneself to study or work*” (4.07%). It should be noted that the strategy of “*Improving Physical Condition, Nutrition and Sleep*” was more prevalent in dentistry students compared to nursing students (*p* < 0.05, 15.1% vs. 6.5%, see previous Table 3).

### 3.6. Short-Term Strategies to Cope with Stress (What Strategy Do You Follow to Immediately (at the Same Time) Deal with Stressful Situations?)

The next two questions focus on two categories of coping strategies for stressful situations: the more immediate and short-term and the more systematic and long-term. Regarding the first category of strategies, the highest percentage of acceptance is gathered from the “*Analysis of The Situation, Staying Cool, Self-Regulation*” (38.70%), which sometimes includes self-animation techniques. This is followed by “*Breathing Techniques*” (32.95%) and other exercises that help to decompress and immediately relax, and the various methods of “*Distraction from The Stressful Situation*” (16.09%), which may include a dedication to work or other activities. Communicating and seeking “*Social/Psychological Support*” gathers acceptance from 14.56%, and seeking free time to engage in “*Hobbies and Recreational Activities*” gathers acceptance from 12.26% of the sample. This is followed by “*Physical Activity*” (8.05%) and “*Organization and Methodicality*” (2.3%).

There was a statistically significant difference between male and female dentistry students’ acceptance of the strategy of “*Social/Psychological Support*”, with female dentistry students presenting a higher acceptance rate (20.0%), compared to male dentistry students (6.0%). No other significant differences were detected between dentistry and nursing departments, in the categories of short-term stress management (see Table 3 and Table 6).

### 3.7. Long-Term Strategies to Cope with Stress (What Do You Usually Do When You Are Stressed for an Extended Period?)

Studying the category of strategies for longer periods of stress, we found that first in order of acceptance ranking comes the “*Analysis of The Situation, Staying Cool*” (31.87%), usually combined with positive thoughts, followed by the search for “*Communication and Social/Psychological Support*” (16.73%) mainly to friends and family or mental health professionals. “*Distraction Combined with Entertainment*” gathers 15.14%, and “*Strategic Thinking and Better Organization of Time*”, 13.15%. This is followed by “*Physical Activity*” (12.35%), “*Dedication to Study*” (11.16%), and focus on “*Personal Time and Personal Care*” (10.76%), which includes, among other things, rest and good nutrition. Another strategy we see at the end is the appeal to one’s “*Spirituality*” (6.77%), which includes techniques such as prayer and meditation.

There was a statistically significant difference between male and female nursing students’ acceptance of the strategy of “*Personal time and Personal Care*”, with female nursing students presenting a lower acceptance rate (8.2%) compared to male nursing students (28.6%). No other significant differences were detected between dentistry and nursing departments relative to long-term stress management (Table 3), yet the “*Patience, Doing Nothing*” strategy was associated with higher levels of stress somatization (ρ = 0.204, *p* < 0.05). Moreover, the “*Analysis of the situation, staying cool, Positive thoughts*” strategy was associated with decreased stress somatization symptom levels (ρ = −0.199, *p* < 0.05) for dentistry students, and the “*Physical Activity and Sports*” strategy with decreased stress somatization levels (ρ = −0.186, *p* < 0.05) for nursing students.

### 3.8. Proposed Interventions for Stress Reduction (What Would You Suggest to Your School Administration to Reduce the Level of Stress You and Your Fellow Students Experience?)

The following are the suggestions of the students for the administration of the school to smooth its malfunctions and alleviate the problems they face in their educational experience, which are perceived as the source of their daily stress. The most accepted and perhaps the most general category of solutions proposed by the students is “*Revision of the educational Program, Better Organization, Staff evaluation*” with proposals to expand it to 5 or 6 years of study, the revision of some courses to focus on more essential and practical knowledge and the better evaluation of the educational staff (45.16%). Another popular suggestion of the students was “*Improved communication and support*”, mainly referring to the professors and supervisors (27.42%), asking for better treatment, greater understanding, and support, especially in their first steps of practical training.

Another important category was “*Reduction of Curriculum Requirements and Hours, Flexibility in Studies*” which includes proposals for easing the studies’ requirements and reducing teaching hours with greater flexibility and breaks (18.95%). Moreover, “*Introduction of Psychological Support Programs*” alongside the educational experience (18.55%) was also accepted by a significant number of students.

Finally, other proposals include the improvement of infrastructure and working conditions, especially in laboratories (5.65%), and better and timelier information from the faculty regarding study programs, laboratories, and exams and the changes that arise from time to time (3.23%).

There was a statistically significant difference between dentistry and nursing students’ acceptance of “*Introduction of Psychological Support Programs*”, with nursing students presenting a higher acceptance rate (24.3%) compared to dentistry students (11.8%). Also, dentistry students supported more strongly, compared to nursing students, the changes of “*Revision of the educational Program, Better Organization, Staff evaluation*” (51.2% vs. 37.4%, *p* < 0.05) and “*Improvement of physical spaces, infrastructure, and working conditions*” (10.2% vs. 1.7%, *p* < 0.05). It should be noted that “*Reduction of Curriculum Requirements and Hours, Flexibility in studies*” was a more prevalent requirement for female dentistry students compared to male dentistry students (26.2% vs. 9.3%, *p* < 0.05) (Table 7).

### 3.9. Need of a Coach (Would You Use the Help of a Coach to Help You Manage Your Anxiety?)

From the responses of the students in the sample, we conclude that the majority is or would be positive about seeking coaching support within the faculty. The main motivation seems to be seeking advice on everyday issues and obviously in managing stress, while a less important motivation seems to be guidance in managing their time, improving their skills, and achieving their goals. Some of the characteristics they look for or would look for in a coach are to show understanding, to have empathy and confidentiality, to be encouraging, but at the same time to be objective and realistic. Regarding the desired frequency with which the students would like to interact with a coach, one session per week gathers the largest percentage (56.45%), with one session per month following (25.81%).

There were no significant differences between male and female participants or between dentistry and nursing departments in the coach acceptance categories (see Table 3). Yet, dentistry students who were willing to accept support from a coach were also experiencing more stress somatization symptoms (ρ = 0.243, *p* < 0.05), while negative attitudes towards coach support were linked with dentistry students reporting lower somatization symptoms (ρ = −0.238, *p* < 0.05). No significant correlations were detected between nursing students’ acceptance of coach support and level of stress somatization symptoms (Table 8).

### 3.10. Other Comments in Open-Ended Questions

In the last open-ended question, students were asked to mention anything they find interesting about the stress they experience. In their answers, we can distinguish two main patterns.

The first concerns the problems and stressful situations that arise in their education/workplace. They find that the very high academic demands for laboratory and clinical performance, the large volume of work, and the speed with which they are required to prepare create a highly stressful situation for them.

*“I am worried about my superiors who, however, do not have the appropriate knowledge to deal with a problem that arises and while they should admit it, they give instructions to their subordinates, who are not sure that it is the right action that they are indicating to be performed.”*

*“As someone who generally struggles in labs, I would like more internships before going into the clinics (e.g., if the dental school had one more year of internships before going into the clinics, I think it would be much better)”*

The situation is aggravated by inadequate infrastructure, non-functional equipment, and insufficient support in critical educational processes. Other factors that increase psychological pressure are the increased responsibility they have in clinical practices and having to deal with real patients and difficult situations very quickly:

*“The anxiety that next year we will enter the clinics, and, in some laboratories, half of the units may not work, as a result of which there is no proper simulation of a real clinical case. I’m getting anxious at the thought that next year I’ll be dealing with a real patient that a mistake of mine could cost him dearly. It also worries me that if, for example, you are sick, it is very difficult to replace a laboratory. Also, if you miss a workshop, it means you are left behind in the next one. And obviously, the amount of reading is huge.”*

*“After my clinical practice, when I leave the hospital, without the clinic staff seeing me, I leave in tears. The conditions are very bad, I get tired both physically and psychologically, I can’t study and I’m worried that I won’t be able to get the educational material in the lessons.”*

Of particular interest is the highlighting of a nursing participant regarding the professional devaluation of nurses in Greece and the role of this devaluation in the psychological burden of nurses.

*“Nurses experience extra stress in Greece because most people don’t appreciate them. I know this is very hard to change. However, I saw that almost all the excellent nursing students continued their studies in another subject. None remained in the infirmary. There are these stereotypes in Greece that anyone who is a nurse is just someone who isn’t good enough to be a doctor. If this doesn’t stop most nurses will never feel fulfilled by their profession.”*

The second pattern of responses focuses on proposed solutions to existing problems and changes they would like to see made in the existing system to reduce stress and improve the academic experience. These focus on improving contact with teaching staff, with the main request being more understanding and support from teachers and teaching staff. More flexibility in academic procedures is also proposed, such as better management of examination times and more opportunities for replacements, but also restructuring of some programs and courses to better prepare for clinical applications, for more practical training, and focus on more essential knowledge. Finally, some student requests focus on strengthening available psychological services and psychological support programs in educational institutions: “*Group therapy, a course that will focus on managing stress in the hospital and during the clinics … I wish we had psychological support. Some students experienced traumatic events, …so if I hadn’t been able to do psychotherapy on my own, I believe I would still be struggling for a long time with my emotions and anxiety*”*.,* “*If there were psychologists at workplaces we could talk about what is troubling us*” *or* “*Due to everyone’s many obligations, it would be good to communicate to resolve questions, but it is also important to be informed in time of the educational material...we also need the textbooks for the courses to read as well as dates for exams ahead of the specific dates*”.

## 4. Discussion

This study investigated stress perceptions, somatization symptoms, and coping strategies among 271 nursing and dentistry undergraduate and postgraduate students from the Department of Dentistry and Nursing at the National and Kapodistrian University of Athens. The study seeks to provide a comprehensive understanding of how stress manifests physically and mentally in these students, how they cope with these challenges, and the need for educational interventions.

### 4.1. Prevalence of Somatization Symptoms Among Dentistry and Nursing Students

The present study examined the prevalence of somatization symptoms among dentistry and nursing students, revealing no statistically significant differences between the two groups, although somatization symptoms were relatively higher in dentistry students. Notably, the findings indicate discipline-specific variations in the severity and nature of somatization symptoms. Dentistry students exhibited a higher prevalence of somatization symptoms, with 41% reporting symptoms compared to 28.20% of nursing students. This aligns with previous research by Feussner et al. (2022) [21], which also found more severe somatization symptoms in dentistry students compared to medical students. Dentistry students’ somatization symptoms were particularly associated with the pressure of meeting daily academic and clinical demands, often linked to performance anxiety and perfectionism inherent in the field of dentistry [72]. In contrast, nursing students tended to experience somatization symptoms related to their inability to manage unexpected and stressful situations, often exacerbated by the emotional and physical demands of patient care [73]. Nursing students frequently encounter high-pressure environments during clinical placements, which can intensify stress levels, leading to physical manifestations such as headaches, gastrointestinal distress, and fatigue [74]. Studies suggest that nursing students may also experience higher levels of burnout and emotional exhaustion [75] due to the empathic nature of their profession and their exposure to critical care scenarios, which might explain the higher somatization rates related to stress [76].

Additionally, the stress somatization patterns differed between the two groups in terms of long-term versus short-term stressors. Dentistry students were more likely to somatize stress related to academic achievement and daily responsibilities [77], possibly due to the highly competitive nature of their program and the demand for precise technical skills [78]. On the other hand, nursing students demonstrated more somatization symptoms related to emotional stress and the unpredictability of patient care, which might be connected to the emotional labor required in nursing, including dealing with patients’ suffering and death [74,79].

Generally, research on somatization symptoms in healthcare students, particularly among nursing and dentistry students, remains severely understudied [80]. This gap in literature is concerning, given the high levels of stress associated with both fields and the potential long-term health implications for students experiencing chronic somatization symptoms as we reported in our study.

### 4.2. Difference in Perceived Stress Levels Between Dentistry and Nursing Students

This study focuses on differences in the perceived stress levels between nursing and dentistry students, particularly in how they somatize stress. Nursing students frequently reported higher stress levels related to the “inability to manage unexpected or difficult situations, insecurity, and panic”. In contrast, dentistry students predominantly experienced stress as “restlessness and psychological pressure”. These variations in stress responses appear to reflect the distinct nature of each discipline and the daily challenges students encounter [81]. Nursing students, for example, often face unpredictable and emotionally charged clinical environments where managing patient care and critical situations can amplify stress [82]. On the other hand, dentistry students are regularly confronted with performance-based stress rooted in the precision and technical skills required in their practice [83]. Further, we could mention that our findings align with previous research, such as a study conducted in Australia that examined perceived stress levels and emotional intelligence among nursing, dentistry, and pharmacy students [75]. This study found a significant negative correlation between emotional intelligence and perceived stress levels in nursing and pharmacy students, suggesting that higher emotional intelligence may help reduce stress. However, perceived stress levels in dentistry and pharmacy students were significantly higher than the normative average and overall higher than in nursing students, as mentioned elsewhere too [76]. These results are consistent with studies focused specifically on dentistry students, which reported moderate [77] to high levels of perceived stress [78,79]. In contrast, studies involving nursing students have generally shown moderate levels of perceived stress, reflecting the different stressors inherent in their training and clinical experiences [27,80]. Overall, these findings prove the importance of addressing discipline-specific stressors in healthcare education programs [84]. Understanding the unique challenges faced by nursing and dentistry students can inform targeted interventions to reduce stress and improve student well-being [40]. Additionally, promoting emotional intelligence and resilience training could be a valuable strategy in reducing the perceived stress levels among students in both fields, as mentioned elsewhere [85].

### 4.3. Somatic Manifestations of Stress

In our study, dentistry and nursing students commonly reported somatic manifestations of stress, such as heart dysphoria, digestive disorders, and headaches, similar to findings elsewhere [9,86]. There were no significant differences between dentistry and nursing students regarding these symptoms, except for temporomandibular joint (TMJ) symptoms, where more females reported issues (24.8%) compared to males (15.1%). This aligns with research that shows the association between stress and TMJ disorders in student populations, where factors like academic pressure and psychosocial stressors contribute to TMJ dysfunction [87,88]. The prevalence of TMJ symptoms among dentistry and nursing students shows their impact on daily activities and quality of life, as documented in studies exploring the relationship between TMJ disorders, stress, and coping mechanisms [89,90,91]. For instance, TMJ disorders can augment during periods of heightened stress, such as during the COVID-19 pandemic, affecting students’ ability to manage academic demands effectively [87]. Effective management strategies for TMJ disorders include stress reduction techniques, ergonomic adjustments, and sometimes pharmacological interventions aimed at alleviating pain and discomfort [92].

Additionally, female participants reported higher rates of tension-type headaches (43.1%) compared to males (18.6%), consistent with findings in nursing and dentistry student populations [87,88,89]. The study also found that headaches significantly impact academic performance and quality of life among health profession undergraduates, aligning with previous research [90,91]. However, our study showed a lower use of medications (2.2%) compared to other self-management strategies reported in the literature, such as over-the-counter medications and relaxation techniques [86,92]. The prevalence of migraine-like headaches is augmented by perceived stress from events like the COVID-19 pandemic, highlighting the need for interventions to manage stress and enhance students’ well-being [93,94].

### 4.4. Demographic Factors Influencing the Experience of Stress-Induced Somatization Symptoms

The present study indicated that stress-induced somatic symptoms are significantly related to gender, with females demonstrating higher levels of stress somatization compared to males. In contrast, factors such as academic year, educational level, and family income did not show statistically significant effects on stress somatization, unlike other findings [46,47,48,49]. From a relevant descriptive cross-sectional study, it was reported that female nursing students who slept six hours or less, did not eat a balanced diet or exercise, attended public universities, had lower grade point averages, intended to leave nursing, and were dissatisfied with the nursing profession reported higher levels of perceived stress. Conversely, nursing students who were satisfied with their field and did not intend to leave it reported lower levels of perceived stress [27]. Also, female nursing students exhibited higher anxiety levels due to multiple factors, including lack of competence and having to deliver bad news [95]. Overall, our findings align with previous research, highlighting gender differences in stress response due to developmental and biological factors [86,96], as well as differences in emotional stress responses related to gender [97].

Furthermore, age differences in emotional responses to daily stress have been documented, with older individuals showing different stress responses compared to younger individuals [98]. This age-related variation in stress response was also evident during the COVID-19 pandemic, where older adults experienced distinct stress levels and life changes impacting their psychological well-being [99]. On the other hand, an observational study involving dentistry students compared first-year and fifth-year students, indicating that older students experienced lower levels of perceived stress and anxiety [100], which was not the case in our study. Similarly, an observational study involving nursing students in a Spanish university revealed that no student experienced high levels of depersonalization or low personal fulfillment, but depersonalization increased as the academic year progressed [96]. Research specific to dentistry students has also shown that the stress response to the dentistry school environment varies by year of study [101]. Older and more advanced students tend to report lower stress levels, suggesting an adaptation or increased coping mechanisms over time [102] but all the above were also not proven on a statistical basis in our study.

### 4.5. Resilience Factors for Dentistry and Nursing Students

In this study, several resilience factors were associated with lower levels of somatization symptoms among both dentistry and nursing students, with emotional balance and self-awareness being particularly significant (34.44%). These findings are consistent with the research by Sperling et al. (2023) [22], which emphasized the role of emotional regulation and self-awareness in reducing somatic symptoms among medical students. Similarly, research has highlighted that self-reflection and emotional self-disclosure contribute to post-traumatic growth in nursing students, further underlining the critical role of self-awareness in cultivating mental health resilience [103].

In line with these findings, studies have also demonstrated that higher levels of empathy, self-awareness, and effective stress management skills predict lower levels of perceived stress and improved well-being among nursing students [27,104]. This is particularly relevant for healthcare students, as their training often involves emotionally challenging situations that demand both clinical expertise and emotional resilience [105]. Rasheed et al. (2021) [106] developed a measure of self-awareness for nurses, showing its significant positive impact on stress reduction and mental health, findings that were echoed by participants in our study. Additionally, research exploring the relationship between spiritual health, resilience, and happiness among dentistry students found that these factors were associated with lower somatization levels and increased overall happiness [107]. Our findings mirror this, as students who reported higher levels of spiritual well-being and resilience also experienced reduced somatic symptoms, suggesting that these factors play a critical role in stress management and mental health for both dentistry and nursing students.

### 4.6. Variation of Coping Strategies in Response to Stress-Induced Somatization Symptoms

In our study, short-term stress management strategies included situational analysis, maintaining composure, self-coercion techniques, self-regulation, breathing exercises, distraction, and social and psychological support. Female dentistry students showed a higher preference (20%) for social and psychological support compared to male students (6%), with no other significant differences observed between dentistry and nursing students [5,108]. Long-term stress management strategies involve situation analysis, maintaining composure, communication, social and psychological support, distraction combined with entertainment, strategic thinking, time management, sleep, physical activity, dedication to study, personal time, self-care (including rest and nutrition), and spirituality such as prayer and meditation, at a lower but significant rate [22]. Dentistry students in our study particularly emphasized the benefits of improved physical well-being, nutrition, and sleep (15.1%) compared to nursing students (6.50%). So, our findings support previous research indicating that regular physical activity and social support contribute to lower rates of somatic symptoms [27].

Moreover, as reported in other studies, alternative ways of stress control, such as prayer, meditation, and yoga, are preferred among healthcare professionals [109,110], as also suggested by our participants. Recent research has highlighted the positive impact of prayer on well-being, emphasizing its dynamic role in daily life. Newman et al. (2023) [111] found that engaging in prayer regularly can significantly enhance an individual’s overall well-being by providing a sense of comfort and purpose. Additionally, it was reported that internal dialogue is a crucial mediator in this relationship, suggesting that prayer facilitates a constructive internal conversation that contributes to improved mental health and emotional stability [112]. Overall, there are insufficient studies in the literature to be associated with our study. Nevertheless, it is generally reported that spirituality is associated with lower levels of depression and stress somatic symptoms in female healthcare students [30,106]. Despite this fact, the present study reported low percentages of spirituality in students’ coping strategies. Possibly, such initiatives could be used as vital components in promoting the psychological well-being of both dentistry and nursing students in future targeted resilient programs where students would be introduced, theoretically and practically, to the positive effects of those strategies [113,114,115,116].

It is also important to mention that significantly elevated levels of stress somatization were observed among participants who adopted a passive approach, characterized by “being patient and doing nothing else”. This passive response to stress contradicts other findings that report that self-guided stress management interventions can effectively reduce stress in college students, highlighting the need for active coping strategies [111,117,118,119]. Furthermore, Bonnesen et al. (2020) [120] emphasized the importance of implementing proactive initiatives to prevent student stress, suggesting that merely waiting for stress to pass without acting can exacerbate somatic symptoms. Some additional studies reported that active coping was supportive of healthcare students’ mental health [116] and was particularly protective of male healthcare students [113,121].

Dentistry students in our study effectively managed stress by employing cognitive strategies such as situational analysis and maintaining a calm demeanor, which correlated with lower levels of stress somatization. This approach aligns with research highlighting the importance of academic resilience and motivational intensity in managing stress [122]. Resilient individuals are known to utilize positive emotions effectively, demonstrating the critical role of emotional regulation in reducing stress [123]. These findings prove the effectiveness of cognitive and emotional strategies in helping dentistry students reduce stress and its somatic effects. Conversely, nursing students achieved similar outcomes by engaging in physical activity and sports to control academic stress. The Mayo Clinic (2024) [124] supports these findings, noting that regular exercise enhances overall health and releases endorphins, which elevate mood and reduce stress, as also mentioned elsewhere [125]. This distinction aligns with broader research on stress coping strategies among students, where effective coping mechanisms in reducing academic stress and fatigue while strengthening self-control are discussed [120].

Furthermore, female nursing students in our study exhibited lower acceptance rates of personal time and personal care (8.20%) compared to male counterparts (28.60%), revealing a gender disparity influenced by societal expectations [47,48,49]. It was reported accordingly that male nursing students often feel less constrained by traditional gender roles, leading to greater acceptance of personal care practices [126]. Gender-defined roles significantly affect nursing students’ attitudes towards self-care, with females prioritizing patient care over personal well-being [127], as was the case in our study too. But self-care is crucial for maintaining overall well-being and professional effectiveness in nursing [128], enhanced by the COVID-19 pandemic’s impact on healthcare professionals’ attitudes towards self-care [129]. Psychological interventions promoting resilience can further enforce self-care practices among nursing students [130], highlighting the need to address gender-specific barriers and cultivate a culture of self-care for their well-being and professional development.

### 4.7. Perceptions of Dentistry and Nursing Students Regarding the Effectiveness of Existing Support Mechanisms in Addressing Somatization Symptoms

This study highlighted dentistry and nursing students’ perceptions of support mechanisms in addressing somatization symptoms, emphasizing areas for improvement such as curriculum revision, faculty organization, and psychological support programs [46,49]. Dentistry students prioritized curriculum changes and better facilities, while nursing students favored psychological support and enhanced educational experiences. Female dentistry students expressed more interest in reducing study requirements [131] and increasing flexibility compared to males. Concerns about supervisor support in demanding conditions were also noted [62,118]. Although direct studies on these mechanisms are lacking, the literature suggests that positive school environments and coping strategies contribute significantly to student health outcomes [129,130,131]. Tailored mental health interventions in dentistry education are also mentioned elsewhere to emphasize the importance of holistic approaches that integrate spiritual and emotional support most needed in these disciplines [66,132,133]. Overall, many participants from the dentistry department pointed out the overwhelming workload and tight deadlines, suggesting the need for a curriculum that allows more flexibility, such as spreading out challenging courses or reducing the frequency of high-stakes assessments [133]. This could help ease the pressure on students and provide a more balanced academic experience [134]. Nursing students, while also concerned with academic pressures, tended to place more emphasis on the need for improved educational experiences, suggesting that enhanced clinical training and practical application of theoretical knowledge would better prepare them for real-world healthcare environments. So, both disciplines could benefit from curriculum reforms that cultivate academic excellence without overburdening students [111,133].

### 4.8. Importance of a Coach in Controlling the Phenomena

In our study, the importance of coaching in managing stress and somatization among dentistry and nursing students was highlighted [35,45,66]. Most students viewed coaching positively, primarily for stress management, as well as for improving time management, skills, and achieving goals [135]. Desired qualities in a coach include understanding, empathy, confidentiality, encouragement, goal orientation, and realism [47,48,49]. Most students preferred weekly sessions (56.45%), while others opted for one to three sessions per month (25.81%). Dentistry students who accepted coaching tended to have higher somatization stress levels, whereas coaching acceptance did not correlate with stress levels among nursing students. Coaching has shown beneficial effects in managing stress and enhancing coping strategies among healthcare students, improving stress management compared to those without coaching [136,137]. Coaching offers personalized support, helping students develop coping mechanisms that address both immediate stress-related concerns and longer-term professional growth [130]. Research has shown that coaching interventions can lead to improvements in mental health, resilience, and academic performance across various fields of healthcare education, including medicine and dentistry [138,139,140]. More specifically, a systematic review protocol by Breslin et al. (2023) highlights the positive effects of coaching on medical student well-being, noting that coaching can reduce distress and enhance overall well-being by providing tailored, one-on-one support [139]. Similarly, Plessas et al. (2022) emphasize the importance of mental health and well-being interventions in the dentistry sector, where students face unique stressors related to both academic and clinical demands, as these interventions not only support students’ mental health but also enhance essential skills such as leadership and emotional resilience [140].

In addition to addressing stress, coaching in medical and dentistry education has been shown to improve critical professional skills. A recent study by Miller-Kuhlmann et al. (2024) [141] offers practical tips for developing coaching programs in medical education, emphasizing how structured coaching can promote leadership, self-management, and independence, particularly in challenging educational environments [141]. Similarly, Aguilar-Ferrándiz et al. (2024) demonstrated that coaching programs designed to enhance empathy and emotional intelligence among health science students resulted in significant improvements in these critical interpersonal skills, further supporting the value of coaching in giving strength to well-rounded healthcare professionals [142]. So overall, implementing a coaching culture within academic settings provides students with essential tools to manage stress effectively while also enhancing long-term resilience and professional skills as we also have proven in our study [141]. This approach is particularly valuable in dentistry, where students not only face academic pressures but also must develop managerial and leadership skills to meet the unique challenges of the field. Coaching interventions, when adapted to the specific challenges faced by students in healthcare, seem to provide a holistic approach to student development that benefits both their mental health and professional growth [142].

### 4.9. Developing Targeted Interventions and Support Mechanisms to Promote the Well-Being of Dentistry and Nursing Students

The findings of this study provide valuable insights for developing targeted interventions and support mechanisms aimed at improving the well-being of dentistry and nursing students. Key recommendations from the students include enhancing better communication with teaching staff, extending internships for practical experience, revising and updating certain courses, and strengthening psychological support services available to students [143]. There is a pressing need to establish more empowered support systems, implement flexible academic policies, and enhance communication between students and educators. Also, effective communication in healthcare education is essential, as strong interpersonal communication not only improves student support but also plays a pivotal role in reducing stress [144]. An interprofessional model that promotes open and transparent communication among healthcare professionals could serve as a valuable framework for students in both disciplines. Such a model would help students manage stress more effectively and improve their overall satisfaction with their educational experience [145,146]. Additionally, there is growing evidence that increased acceptance of coaching programs correlates positively with better stress management outcomes [147]. Our findings support the importance of providing specific training in resilience-building techniques, such as stress and time management [135] and emotional regulation strategies, to enhance students’ coping skills and overall mental health [47,48,49,132,148].

So, practically, future implementations of our findings include offering stress management workshops tailored to healthcare students’ needs and incorporating techniques such as mindfulness, relaxation exercises, and time management strategies to help manage academic pressure and emotional stress [135,148]. Additionally, the establishment of peer support networks or mentorship programs, which would allow students to connect with senior peers, providing emotional support and shared coping strategies for academic and clinical stress, will also be effective [149]. We should further propose integrating coping skills training into the healthcare curriculum, with brief sessions focused on developing resilience and effective stress management techniques [150,151,152]. Furthermore, it is also recommended to provide faculty and staff training on stress awareness to help educators recognize students in distress and offer appropriate referrals to support services [131,153]. Enhancing physical and mental health support services, including counseling, stress-relief programs, and access to physiotherapy or dental care for TMJ disorders, will also be crucial for students experiencing physical manifestations of stress [154,155,156]. Finally, we emphasize the importance of resilience-building initiatives, such as workshops on emotional regulation and problem-solving, which can equip students with tools to better manage their academic and clinical workloads, thus reducing the likelihood of somatization symptoms [157,158,159]. These concrete approaches derived from our data are supported by current literature and also support students’ well-being and resilience in both disciplines.

### 4.10. Limitations and Strengths of the Study

This study has several acknowledged limitations. Firstly, while the sample size is adequate for statistical analysis, it is confined to nursing and dentistry students from a single institution, limiting its generalizability to students in other disciplines or institutions. At the same time, it provides valuable insights into stress in healthcare education, particularly within the Greek context. As such, these findings should be considered within the framework of the Greek academic environment, where cultural and educational factors may influence how students experience and manifest stress. Despite this limitation, the insights gained can still offer meaningful contributions to the broader discourse on stress and coping in healthcare students as we proved that it is aligned with multiple other studies. Of course, future research could build on this by examining larger, more diverse student populations across different cultural contexts to enhance the generalizability of the results.

Secondly, reliance on self-reported data introduces potential biases such as social desirability and recall bias [160,161]. Additionally, the qualitative analysis of open-ended questions, while detailed and carefully structured, may be subject to researcher bias in categorizing responses since it does not account for confounding factors like personality traits, pre-existing mental health conditions, or external stressors beyond the academic environment [162,163,164]. Further, the lack of longitudinal data limits the understanding of how stressful perceptions and coping strategies evolve over students’ academic careers. Methodological triangulation was also not explicitly employed in this study. Instead, the research focused on utilizing survey instruments to gather data on perceived stress and associated symptoms as mentioned elsewhere [10,17,56]. So, future research could benefit from incorporating qualitative methods or multiple data sources to triangulate findings and enhance the depth of understanding regarding stress experiences and coping mechanisms among dentistry and nursing students [165,166,167,168]. Finally, a last potential limitation of this study is the gender imbalance among participants, which may affect the generalizability of the findings related to stress somatization. Since gender differences in stress responses are well-documented [169,170], a more balanced gender distribution would have provided a clearer comparison of stress somatization patterns between male and female participants. Future studies should aim for a more equal gender representation to ensure that the findings accurately reflect gender-specific stress and somatization experiences.

Despite the acknowledged limitations, this study provides valuable insights into stress, somatization, and coping strategies among dentistry and nursing students. While the sample size is limited to a single institution, it aligns with other focused studies on specific student populations, where larger samples are challenging due to the niche nature of the subject [171,172]. Also, the carefully selected participants from both dentistry and nursing programs offer a relevant perspective on discipline-specific stressors, informing targeted educational and mental health interventions as mentioned elsewhere [173]. Further, cross-sectional design, often viewed as a limitation, is a strength in this context [174]. It provides us with a timely snapshot of stress and coping dynamics among healthcare students, which is crucial in a rapidly changing educational environment, especially after global disruptions like the pandemic. As it is reported, cross-sectional studies are effective for identifying prevalence rates and exploring variable relationships, serving as a foundation for more in-depth, longitudinal research [175,176]. So, it is suggested that this baseline information is essential for future studies, policy development, and educational practices [177]. Our study also utilized validated scales to quantitatively assess stress levels, coping strategies, and resilience among students, revealing significant discipline-specific differences [178]. These quantitative findings are enriched by qualitative insights from open-ended responses, offering a deeper context to the students’ stress experiences [179]. Combining these methods aligns with the mixed-methods framework, providing a comprehensive understanding of the research problem and laying the groundwork for more targeted future studies [180,181,182,183,184,185,186,187,188,189,190].

Moreover, descriptive research, as employed in this study, plays a crucial role in underexplored areas such as stress and coping strategies in healthcare education. It helps identify key trends and associations, which are foundational for developing hypothesis-driven research [60,189,190]. Our findings emphasize that while common issues exist across healthcare disciplines, significant differences between dentistry and nursing students call for tailored approaches to support student well-being and academic success. In our future approach, we plan to address limitations by utilizing larger, more diverse samples, employing longitudinal designs, and considering additional variables impacting student stress and coping mechanisms.

## 5. Conclusions

This study highlights gender and departmental differences in stress perception, somatization, and coping strategies among nursing and dentistry students. Female students reported higher stress somatization levels. Stress interpretations varied, with major themes including insecurity, psychological pressure, and physical symptoms. Nursing students associate stress with insecurity and experience higher somatization, while dentistry students link stress to daily obligations and goals. Coping strategies focused on emotional balance, self-awareness, and stress management, with immediate techniques involving situational analysis and breathing, and long-term strategies including maintaining calm and seeking support. The study reveals academic pressures and inadequate infrastructure as key stress contributors, emphasizing the need for improved support systems, flexible academic procedures, and better communication. High acceptance of coaching support was linked to better stress management outcomes.

## Figures and Tables

**Table 1:** Percentage distribution according to demographic profiles.

		Total (N = 271)		Dental Department (n = 145, 53.5%)		Nursing Department (n = 126, 46.5%)		χ^2^ Test Results
		N	%	N	%	N	%	
Gender	Male	85	31.40%	54	37.30%	31	24.60%	χ^2^(1) = 5, *p* = 0.025
	Female	186	68.60%	91	62.80%	95	75.40%	
Educational level	Postgraduate studies/PhD	54	19.90%	3	2.10%	51	40.50%	χ^2^(1) = 62.33, *p* < 0.001
	Undergraduate studies	217	80.10%	142	97.90%	75	59.50%	
Year of studies	1st year	12	4.3%	5	3.4%	7	5.3%	χ^2^(1) = 62.17, *p* < 0.001
	2nd year	18	6.5%	7	4.8%	11	8.3%	
	3rd year	64	23.1%	43	29.7%	21	15.9%	
	4th year	52	18.8%	18	12.4%	34	25.8%	
	5th year	71	25.6%	69	47.6%	2	1.5%	
	Postgraduate students	60	21.7%	3	2.1%	57	41.7%	
Family income	<EUR 15,000	32	16.20%	14	14.60%	18	17.80%	χ^2^(1) = 24.71, *p* < 0.001
	EUR 15,001–25,000	71	36.00%	24	25.00%	47	46.50%	
	EUR 25,001–35,000	37	18.80%	15	15.60%	22	21.80%	
	EUR 35,001–50,000	34	17.30%	24	25.00%	10	9.90%	
	>EUR 50,000	23	11.70%	19	19.80%	4	4.00%	

**Table 2:** Stress somatization for students of different departments and genders.

Stress Somatization *		Department					
		Total (N = 271)		Dentistry (n = 145, 53.5%)		Nursing (n = 126, 46.5%)	
		M	SD	M	SD	M	SD
Gender	Male (n = 85, 31.4%)	7.94_A_	6.14	8.23 _a_	6.37	7.48 _a_	5.80
	Female (n = 186, 68.6%)	10.22_B_	5.23	10.90 _a_	5.46	9.59 _a_	4.95
	Total (Ν = 271)	9.51	5.62	9.92	5.93	9.06	5.23

Note: Values in the same row or the same column not sharing the same subscript are significantly different at *p* < 0.05 in the two-sided independent samples *t*-tests. * Minimum = 0, maximum = 28.

**Table 3:** Differences in stress somatization symptoms by gender.

		Gender					
		Total		Male		Female	
		N	%	N	%	N	%
1. Do you experience pain in the facial area during the day and/or night?	Never, not applicable	139	50.4%	51 _a_	59.3%	88 _a_	46.3%
	Usually not applicable	64	23.2%	17 _a_	19.8%	47 _a_	24.7%
	Not sure	35	12.7%	8 _a_	9.3%	27 _a_	14.2%
	Very true	31	11.2%	7 _a_	8.1%	24 _a_	12.6%
	Always applies	7	2.5%	3 _a_	3.5%	4 _a_	2.1%
2. Do you hear a sound from the temporomandibular joint during movements of the mandible?	Never, not applicable	131	47.5%	49 _a_	57.0%	82 _b_	43.2%
	Usually not applicable	41	14.9%	7 _a_	8.1%	34 _b_	17.9%
	Not sure	44	15.9%	17 _a_	19.8%	27 _a_	14.2%
	Very true	32	11.6%	6 _a_	7.0%	26 _a_	13.7%
	Always applies	28	10.1%	7 _a_	8.1%	21 _a_	11.1%
3. Do you clench or grind teeth during the night and/or day?	Never, not applicable	91	33.0%	33 _a_	38.4%	58 _a_	30.5%
	Usually not applicable	35	12.7%	11 _a_	12.8%	24 _a_	12.6%
	Not sure	51	18.5%	14 _a_	16.3%	37 _a_	19.5%
	Very true	54	19.6%	15 _a_	17.4%	39 _a_	20.5%
	Always applies	45	16.3%	13 _a_	15.1%	32 _a_	16.8%
4. Do you experience headaches?	Never, not applicable	52	18.8%	30 _a_	34.9%	22 _b_	11.6%
	Usually not applicable	64	23.2%	25 _a_	29.1%	39 _a_	20.5%
	Not sure	62	22.5%	15 _a_	17.4%	47 _a_	24.7%
	Very true	57	20.7%	7 _a_	8.1%	50 _b_	26.3%
	Always applies	41	14.9%	9 _a_	10.5%	32 _a_	16.8%
5. Is your sleep disturbed?	Never, not applicable	63	22.8%	26 _a_	30.2%	37 _b_	19.5%
	Usually not applicable	74	26.8%	20 _a_	23.3%	54 _a_	28.4%
	Not sure	61	22.1%	15 _a_	17.4%	46 _a_	24.2%
	Very true	51	18.5%	17 _a_	19.8%	34 _a_	17.9%
	Always applies	27	9.8%	8 _a_	9.3%	19 _a_	10.0%
6. Do you feel nervous-or/annoyed-at your relationships in the student/work environment?	Never, not applicable	58	21.0%	33 _a_	38.4%	25 _b_	13.2%
	Usually not applicable	60	21.7%	17 _a_	19.8%	43 _a_	22.6%
	Not sure	67	24.3%	13 _a_	15.1%	54 _b_	28.4%
	Very true	65	23.6%	18 _a_	20.9%	47 _a_	24.7%
	Always applies	26	9.4%	5 _a_	5.8%	21 _a_	11.1%
7. Are you taking medication to calm down from the responsibilities of everyday life?	Never, not applicable	236	85.5%	73 _a_	84.9%	163 _a_	85.8%
	Usually not applicable	18	6.5%	3 _a_	3.5%	15 _a_	7.9%
	Not sure	10	3.6%	3 _a_	3.5%	7 _a_	3.7%
	Very true	6	2.2%	5 _a_	5.8%	1 _a_	0.5%
	Always applies	6	2.2%	2 _a_	2.3%	4 _a_	2.1%

Note: Values in the same row and subtable not sharing the same subscript are significantly different at *p* < 0.05 in the two-sided chi-square test.

**Table 4:** Categories/themes extracted from open-ended questions relative to perceptions of stress, stress management strategies and requirements, by students’ department and gender.

	Total Sample		Dental/Total		Nursing/Total		Dental/Male		Dental/Female		Nursing/Male		Nursing/Female	
	N	%	N	%	N	%	N	%	N	%	N	%	N	%
*What does “stress” mean to you?*														
Physical symptoms	54	19.9%	26	18.7%	26	21.0%	7	13.7%	19	21.6%	6	18.8%	20	21.7%
Restlessness and psychological pressure	93	34.3%	57 _a_	41.0%	35 _b_	28.2%	23	45.1%	34	38.6%	9	28.1%	26	28.3%
Difficulty concentrating, Disorganization of thoughts and feelings, Numbness	48	17.7%	26	18.7%	20	16.1%	9	17.6%	17	19.3%	8	25.0%	12	13.0%
Obsessive thoughts	26	9.6%	17	12.2%	8	6.5%	6	11.8%	11	12.5%	2	6.3%	6	6.5%
Insecurity, Inability to handle unexpected or difficult situations, Panic	104	38.4%	48	34.5%	52	41.9%	17	33.3%	31	35.2%	15	46.9%	37	40.2%
Fear of failure, lack of confidence	18	6.6%	10	7.2%	8	6.5%	4	7.8%	6	6.8%	2	6.3%	6	6.5%
Pressure to meet daily obligations/long-term goals	26	9.6%	11	7.9%	15	12.1%	5	9.8%	6	6.8%	2	6.3%	13	14.1%
*How and where in the body do you experience stress?*														
Heart or chest discomfort	107	41.8%	54	41.2%	50	42.7%	22	46.8%	32	38.1%	10	34.5%	40	45.5%
Digestive Disorders, Anorexia	106	41.4%	48	36.6%	53	45.3%	17	36.2%	31	36.9%	14	48.3%	39	44.3%
Breathing difficulties, Cough	30	11.7%	14	10.7%	15	12.8%	4	8.5%	10	11.9%	3	10.3%	12	13.6%
Headaches, Nausea	93	36.3%	44	33.6%	48	41.0%	18	38.3%	26	31.0%	11	37.9%	37	42.0%
General muscle tension and discomfort	28	10.9%	13	9.9%	13	11.1%	7	14.9%	6	7.1%	4	13.8%	9	10.2%
Dental symptoms	19	7.4%	12	9.2%	7	6.0%	3	6.4%	9	10.7%	3	10.3%	4	4.5%
Sweating, itching and skin diseases	27	10.5%	14	10.7%	11	9.4%	5	10.6%	9	10.7%	5	17.2%	6	6.8%
*How do you feel you can improve your resilience?*														
Dedication to work or study	11	4.1%	4	2.9%	7	5.7%	1	2.0%	3	3.4%	0	0.0%	7	7.5%
Psychotherapy and psychological support	47	17.4%	23	16.5%	20	16.3%	7	14.0%	16	18.0%	4	13.3%	16	17.2%
Managing stressors	70	25.9%	31	22.3%	39	31.7%	14	28.0%	17	19.1%	14	46.7%	25	26.9%
Planning-time management	25	9.3%	9	6.5%	16	13.0%	2	4.0%	7	7.9%	3	10.0%	13	14.0%
Improving physical condition, nutrition and sleep	29	10.7%	21_a_	15.1%	8 _b_	6.5%	7	14.0%	14	15.7%	2	6.7%	6	6.5%
Emotional balance and self-awareness	93	34.4%	47	33.8%	43	35.0%	15	30.0%	32	36.0%	12	40.0%	31	33.3%
Social support and socialization	20	7.4%	10	7.2%	8	6.5%	2	4.0%	8	9.0%	1	3.3%	7	7.5%
I don’t know	22	8.1%	14	10.1%	8	6.5%	7	14.0%	7	7.9%	1	3.3%	7	7.5%
*What strategy do you follow to immediately (at the same time) deal with stressful situations?*														
Breathing and relaxation techniques	86	33.0%	45	33.3%	40	33.6%	15	30.0%	30	35.3%	8	28.6%	32	35.2%
Distraction and thought management	42	16.1%	19	14.1%	21	17.6%	6	12.0%	13	15.3%	3	10.7%	18	19.8%
Analysis of the situation, staying cool, self-regulation	101	38.7%	53	39.3%	47	39.5%	21	42.0%	32	37.6%	11	39.3%	36	39.6%
Organization and methodicality	6	2.3%	5	3.7%	1	0.8%	1	2.0%	4	4.7%	0	0.0%	1	1.1%
Physical activity	21	8.0%	9	6.7%	11	9.2%	2	4.0%	7	8.2%	4	14.3%	7	7.7%
Talking with Others and Social/Psychological Support	38	14.6%	20	14.8%	16	13.4%	3 _a_	6.0%	17 _b_	20.0%	4	14.3%	12	13.2%
Hobbies, entertainment and leisure	32	12.3%	13	9.6%	18	15.1%	7	14.0%	6	7.1%	6	21.4%	12	13.2%
*What do you usually do when you are stressed for a long period* (e.g., *during an exam*)														
Patience/Nothing	38	15.1%	18	13.8%	20	17.7%	4	8.7%	14	16.7%	4	14.3%	16	18.8%
Distraction and entertainment	80	31.9%	39	30.0%	38	33.6%	13	28.3%	26	31.0%	9	32.1%	29	34.1%
Analysis of the situation, staying cool, positive thoughts	31	12.4%	17	13.1%	12	10.6%	9	19.6%	8	9.5%	3	10.7%	9	10.6%
Physical exercise and sports	42	16.7%	25	19.2%	16	14.2%	6	13.0%	19	22.6%	7	25.0%	9	10.6%
Communication, psychological/social support	27	10.8%	14	10.8%	13	11.5%	5	10.9%	9	10.7%	3	10.7%	10	11.8%
Personal time and Personal Care	33	13.1%	16	12.3%	15	13.3%	6	13.0%	10	11.9%	8 _a_	28.6%	7 _b_	8.2%
Strategic thinking and organization	28	11.2%	18	13.8%	9	8.0%	7	15.2%	11	13.1%	1	3.6%	8	9.4%
Dedication to study	17	6.8%	6	4.6%	11	9.7%	3	6.5%	3	3.6%	2	7.1%	9	10.6%
Spirituality	5	2.0%	4	3.1%	1	0.9%	2	4.3%	2	2.4%	0	0.0%	1	1.2%
*What would you suggest to your school administration to reduce the level of stress you and your fellow students experience?*														
Reduction of curriculum requirements and hours, flexibility in studies	47	18.9%	26	20.5%	21	18.3%	4 _a_	9.3%	22 _b_	26.2%	4	12.9%	17	20.2%
Introduction of psychological support programs	46	18.5%	15 _a_	11.8%	28 _b_	24.3%	4	9.3%	11	13.1%	4	12.9%	24	28.6%
Revision of the educational program, better organization, staff evaluation	112	45.0%	65 _a_	51.2%	43 _b_	37.4%	21	48.8%	44	52.4%	13	41.9%	30	35.7%
Better and more timely information about the curriculum/labs/exams and the resulting changes	8	3.2%	4	3.1%	3	2.6%	1	2.3%	3	3.6%	1	3.2%	2	2.4%
More practice, better preparation	14	5.6%	11 _a_	8.7%	3 _b_	2.6%	3	7.0%	8	9.5%	0	0.0%	3	3.6%
Improvement of physical spaces, infrastructure and working conditions	15	6.0%	13 _a_	10.2%	2 _b_	1.7%	6	14.0%	7	8.3%	0	0.0%	2	2.4%
Improved communication and support, Better treatment by teachers and supervisors	68	27.3%	40	31.5%	26	22.6%	16	37.2%	24	28.6%	6	19.4%	20	23.8%
*Would you use the help of a coach to help you manage your anxiety?*														
Positive about support from a coach	141	53.8%	70	52.2%	68	56.7%	22	45.8%	48	55.8%	15	48.4%	53	59.6%
Negative about support from a coach	106	40.5%	57	42.5%	44	36.7%	24	50.0%	33	38.4%	15	48.4%	29	32.6%
Preference for support from mental health counselors	18	6.9%	9	6.7%	9	7.5%	3	6.3%	6	7.0%	1	3.2%	8	9.0%

Note: Values in the same row and subtable not sharing the same subscript are significantly different at *p* < 0.05 in the two-sided chi-square test.

**Table 5:** Spearman correlations between the categories of meaning and manifestation of stress and stress somatization symptoms, by students’ department. The method was applied only to the quantitative data, not the qualitative themes.

	Somatization Symptoms	
	Dentistry	Nursing
*What does* “*stress*” *mean to you?*		
Physical symptoms	−0.099	−0.093
Restlessness and psychological pressure	−0.055	0.03
Difficulty concentrating, disorganization of thoughts and feelings, Numbness	−0.029	0.066
Obsessive thoughts	0.037	0.01
Insecurity, inability to handle unexpected or difficult situations, Panic	0.032	0.276 *
Fear of failure, lack of confidence	−0.114	−0.074
Pressure to meet daily obligations/long-term goals	0.187 *	−0.152
*How and where in the body do you experience stress?*		
Heart or chest discomfort	−0.089	0.036
Digestive disorders, anorexia	−0.011	0.002
Breathing difficulties, cough	−0.035	0.054
Headaches, nausea	−0.005	−0.103
General muscle tension and discomfort	−0.06	−0.016
Dental symptoms	0.106	0.059
Sweating, itching and skin diseases	−0.073	0.027

* Statistical significance between the two departments.

**Table 6:** Spearman correlations between the categories stress management strategies and stress somatization symptoms, by students’ department.

	Somatization Symptoms	
	Dentistry	Nursing
*How do you feel you can improve your resilience?*		
Dedication to work or study	0.109	−0.038
Psychotherapy and psychological support	0.127	0.059
Managing stressors	0.006	−0.065
Planning-time management	0.090	−0.038
Improving physical condition, nutrition and sleep	−0.112	−0.060
Emotional Balance and self-awareness	−0.059	0.061
Social support and socialization	−0.111	−0.080
I don’t know	−0.091	−0.013
*What strategy do you follow to immediately (at the same time) deal with stressful situations?*		
Breathing and relaxation techniques	0.037	−0.051
Distraction and thought management	−0.001	0.008
Analysis of the situation, staying cool, self-regulation	−0.154	−0.075
Organization and methodicality	0.120	−0.083
Physical activity	0.018	−0.054
Social/Psychological support	0.134	−0.074
Hobbies and recreational activities	−0.046	0.046
*What do you usually do when you are stressed for a long period?*		
Patience/Nothing	0.204 *	0.058
Distraction and entertainment	0.138	−0.08
Analysis of the situation, staying cool, Positive thoughts	−0.199 *	0.153
Physical activity and sports	0.06	−0.186 *
Communication, Psychological/Social Support	−0.023	0.000
Personal time and Personal care	−0.112	−0.015
Strategic thinking and organization	−0.166	−0.025
Dedication to study	−0.028	−0.029
Spirituality	−0.005	−0.087

* Statistical significance between the two departments.

**Table 7:** Spearman correlations between suggestions to school administration for better stress management support and stress somatization symptoms by students’ department.

What Would You Suggest to Your School Administration to Reduce the Level of Stress You and Your Fellow Students Experience?	Somatization Symptoms	
	Dentistry	Nursing
Reduction of Curriculum Requirements and Hours, Flexibility in studies	0.130	0.005
Introduction of Psychological Support Programs	0.101	−0.183
Revision of the educational Program, Better Organization, Staff evaluation	0.086	0.012
Better and more timely information about the curriculum/labs/exams and the resulting changes	−0.038	0.097
More practice, better preparation	0.030	0.049
Improvement of physical spaces, infrastructure, and working conditions	−0.081	−0.008
Improved communication and support, better treatment by teachers and supervisors	0.050	0.073

**Table 8:** Spearman correlations between the categories of coach acceptance and somatization symptoms by students’ department.

	Somatization Symptoms	
	Dentistry	Nursing
Positive about support from a coach	0.243 **	−0.077
Negative about support from a coach	−0.238 **	0.067
Preference for support from mental health counselors	0.059	0.026

** Significant correlation between stress somatization symptoms and need for a coach.

## Data Availability

The original contributions presented in the study are included in the article; further inquiries can be directed to the corresponding authors.

## Appendix A

Introductory message

ONLY FOR UNDERGRADUATE DENTAL and NURSING STUDENTS

The questionnaire of this study includes a general part (A) with demographic data, part (B) referring TMJ disorders due to stress and part C includes open-ended questions exploring participants’ views on stress factors, somatization of stress in the body, strategies followed to control stress and need for educational interventions.

The survey is designed to be anonymous, ensuring that no personal information is collected during the process. Participation is voluntary and no reward of any kind is provided for your participation. You can fill in the questionnaire ONLY once. Completing the questionnaire implies acceptance of the personal data protection rules. The results of this study will be used for scientific publications and actions to improve working conditions in the departments involved. For observations, comments, and clarifications you can contact the research group.

## Appendix B

**Questionnaire**

**PART ONE**

Q1. What is your gender?

Q2. What is your level of education (1- 5 years, degree/diploma) undergraduate, postgraduate, PhD, postdoctoral)

Q3. What is your place of origin?

Athens

Other urban center (capital of a prefecture)

Continental region (towns and villages)

Island region (towns and villages)

Other country outside Greece

Q4. Which dental specialty would you like to pursue in the future? (for dental students)

General dentistry

Pedodontics

Orthodontics

Oral surgery

Dentistry-sensitive dentistry

Endodontics

Periodontology

Stomatology/hospital dentistry

Prosthetics-implants

I will not practice dentistry

I am not a dental student

Q5. Which nursing specialization would you like to pursue? (for nursing students)

Community nursing

Psychiatric nursing

Surgical nursing

Pathological nursing

Emergency nursing

Oncology nursing

I will not practice nursing

I am not a nursing student

Q6. What is your family’s annual income?

P1. less than 15.000 €

Ρ2. 15.001-25.000 €

Ρ3. 25.001-35.000 €

Ρ4. 35.001-50.000 €

P5. more than € 50.000

Ρ6. I do not know

Ρ7. I have never worried about this issue

Q7.1. What are you thinking of doing after graduation? (for dental students)

Ρ1. I will open my own clinic

Ρ2. I will work as a clerk in a colleague’s office for a few years until I see what I can do

Ρ3. I will work as a dentist abroad

Ρ4. I will do postgraduate studies and work in a colleague’s office at the same time

Ρ5. I will not practice clinical dentistry but will do something else in the field

Ρ6. I will do something else professionally

Ρ7. I will continue my studies in another discipline

Ρ8. I am not a student of dentistry

Q7.2 What do you plan to do when you graduate? (for nursing students)

Ρ1. I will work in a private clinic

Ρ2. I will work in a public hospital

Ρ3. I will do postgraduate studies

Ρ4. I will do something else professionally

Ρ5. I will continue my studies in another science

Ρ6. I am not a nursing student

**PART TWO**

This part of the study collects data on the somatization of stress and its effect on students’ SGD issues. It will be answered on a scale:

0. = Never, not applicable

1. = Usually not applicable

2. = Applies and does not apply

3. = Very true

4. = Always applies

Q8. Do you experience pain in the facial area during the day and/or night?

Q9. Do you hear a sound from the temporomandibular joint during movements of the mandible?

Q10. Do you clench or grind teeth during the night and/or day?

Q11. Do you experience headaches?

Q12. Is your sleep disturbed?

Q13. Do you feel nervous-or/annoyed-at your relationships in the student/work environment?

Q14. Are you taking medication to calm down from the responsibilities of everyday life?

**PART THREE**

The third part includes open-ended questions aimed at gathering desired actions and initiatives intended for the management and handling of stress, as well as the improvement of resilience.

Q15. What does stress mean to you?

Q16. How and where in your body do you experience stress?

Q17. What does resilience mean to you?

Q18. How do you think you can improve your resilience?

Q19. What strategy/strategies do you follow to deal with stressful situations?

Q20. What do you usually do when you are stressed?

Q21. What would you suggest to your school administration to reduce the level of stress you and your fellow students experience?

Q22. Do you consider psychological resilience to be innate, or can it be cultivated?
